# On Anchylosis of the Lower Jaw

**Published:** 1852-04

**Authors:** 


### ARTICLE XII.

### *On Anchylosis of the Lower Jaw.* By Dr. Wernher.

S. R——, æt. twenty-three, when three years of age underwent severe salivation, after which the jaw remained in a fixed state. Notwithstanding the absence of masticatory power, he was well nourished. The jaw was quite immovable, firm pressure or traction exerting no effect upon the position of the teeth. The incisors and molars were indeed, for the most part, wanting, the roots of such as did exist, projecting beyond the alveoli of the diminutive jaw-bone. The jaws were so far separated, that with some trouble, the little finger could be introduced in front; but from the anterior molars on each side backwards, bony arches connected the upper and lower jaws. The buccal mucous membrane was attached to the gums at the anterior edges of these arches, but the temporal and masseter muscles remained free. Speech much resembled that which takes place with the mouth closed; and food which did not require mastication was introduced between the defective teeth. To remedy this state of things, the gums were separated by an incision from the cheeks and lips, and a broad portion of the connecting arch on either side removed by a small saw. The jaws could now be expanded, by aid of a mouth speculum, to the extent of half an inch, some painful stretching of the muscles being induced. The patient was enabled, too, to voluntarily close the mouth again, proving that twenty years of inactivity had not destroyed the functions of the joints and muscles. After several weeks perseverance in gradual dilatation, a still wider expansion was obtained, enabling the patient to chew food that was not too hard, which, indeed, the loose state of his teeth also prevented him biting.

Anchylosis of the lower jaw may occur in three localities. 1st. The head of the condyle may become fixed in its glenoid cavity. This is the most frequent form, examples of which are recorded by Sandifort, Blandin, Cruveilhier, Howship, Holscher, Hyrtl, &c. 2d. The coronoid process may become attached to the zygomatic arch. Of this, but two observations are recorded, one by Sandifort (Museum. Anat. vol. iv,) and by Sebastian in his "Essay on Anchylosis," published at Groningen, 1826. 3d. The alveolar processes may become conjoined. Of this, there are three examples on record, besides the one now narrated, which are to be found related in Walther's "Museum Anat." in "Rust's Magazin," Band 1, and in Bonnet's (cited from Kunholz) "Maladies des Articulations."

*Am. Journal of Med. Sciences.*

